# Analysis of the Effectiveness of Metaheuristic Methods on Bayesian Optimization in the Classification of Visual Field Defects

**DOI:** 10.3390/diagnostics13111946

**Published:** 2023-06-02

**Authors:** Masyitah Abu, Nik Adilah Hanin Zahri, Amiza Amir, Muhammad Izham Ismail, Azhany Yaakub, Fumiyo Fukumoto, Yoshimi Suzuki

**Affiliations:** 1 Center of Excellence for Advance Computing, Faculty of Electronic Engineering & Technology, Universiti Malaysia Perlis, Kangar 01000, Malaysia; 2 Institute of Engineering Mathematics, Faculty of Applied and Human Sciences, Universiti Malaysia Perlis, Arau 02600, Malaysia; 3 Department of Ophthalmology & Visual Science, Universiti Sains Malaysia, Kubang Kerian 16150, Malaysia; 4 Graduate Faculty of Interdisciplinary Research, University of Yamanashi, Kofu 400-0016, Japan

**Keywords:** metaheuristic method, Bayesian optimization, acquisition function, VGGNet, visual field defect

## Abstract

Bayesian optimization (BO) is commonly used to optimize the hyperparameters of transfer learning models to improve the model’s performance significantly. In BO, the acquisition functions direct the hyperparameter space exploration during the optimization. However, the computational cost of evaluating the acquisition function and updating the surrogate model can become prohibitively expensive due to increasing dimensionality, making it more challenging to achieve the global optimum, particularly in image classification tasks. Therefore, this study investigates and analyses the effect of incorporating metaheuristic methods into BO to improve the performance of acquisition functions in transfer learning. By incorporating four different metaheuristic methods, namely Particle Swarm Optimization (PSO), Artificial Bee Colony (ABC) Optimization, Harris Hawks Optimization, and Sailfish Optimization (SFO), the performance of acquisition function, Expected Improvement (EI), was observed in the VGGNet models for visual field defect multi-class classification. Other than EI, comparative observations were also conducted using different acquisition functions, such as Probability Improvement (PI), Upper Confidence Bound (UCB), and Lower Confidence Bound (LCB). The analysis demonstrates that SFO significantly enhanced BO optimization by increasing mean accuracy by 9.6% for VGG-16 and 27.54% for VGG-19. As a result, the best validation accuracy obtained for VGG-16 and VGG-19 is 98.6% and 98.34%, respectively.

## 1. Introduction

Deep learning (DL) is a subfield of machine learning (ML) involving the training of artificial neural networks to automatically learn complex features and representations of the input data, allowing them to perform various tasks such as image and speech processing from large amounts of data [1]. Transfer learning utilizes a previously trained neural network model to improve the training of a new deep learning model on a related task [2,3]. The pre-trained model is adapted or fine-tuned using less training data for the new task or problem. Transfer learning can be beneficial when there is insufficient training data or when training a new model is time-consuming or computationally costly.

The error of a deep learning model can be minimized by selecting the optimal set of hyperparameters to maximize the model’s performance on the validation set. This optimal set of hyperparameters can be retrieved efficiently and effectively through optimization to achieve high performance in a particular task [4]. In DL, optimization significantly enhances the model’s performance by minimizing the errors or maximizing accuracy as an objective function during training. In the case of transfer learning, optimization is applied to fine-tune a previously trained model to improve a new related task.

Many previous works have proposed Bayesian Optimization (BO) [5,6,7,8] as an optimization method that uses a probabilistic model of the objective function. This probabilistic model, also called a surrogate model, searches the optimal set of hyperparameters and predicts the model’s performance at untested points in the hyperparameter space. The performance of the surrogate model will be evaluated at different parameter settings by maximizing the Expected Improvement (EI) of the surrogate model. First, the EI is measured to identify how much the objective function is expected to improve the model’s performance, or in other words, it measures the potential gain that can be obtained by evaluating the model at a new set of hyperparameters. Then, the acquisition function in BO is used to determine which set of hyperparameters should be evaluated next by balancing the trade-off between exploring regions of the search space where little is known and exploiting regions where the objective function is believed to be optimal [9]. However, the repeated process of evaluating the acquisition function in BO and updating the surrogate model can cause the computational cost to become prohibitively expensive due to increasing dimensionality, making it more challenging to achieve the global optimum.

In recent years, DL and transfer learning have become popular image classification techniques due to their high performance and ability to learn complex features from large amounts of data, and BO has grown in popularity as an optimization method. Previously, Shankar et al. [10] proposed a hyperparameter tuning of the BO framework to deal with the hyperparameter estimation problem in ML for diabetic retinopathy classification. They evaluate the performance of the CNN model with different hyperparameter settings, including batch size, learning rate, number of epochs, and weight decay. On the other hand, Hung et al. [9] have discussed contextual bandit problems, specifically in EI Contextual bandit problems involve making a decision based on contextual information with the goal of maximizing a reward for BO. From the experiment results, their proposed method outperformed other existing contextual bandits in Upper Confidence Bound (UCB) and Thompson Sampling (TS) by achieving a cumulative reward. Another related work was conducted by Abu et al. [11]. The authors discussed a thorough analysis of the transfer learning method when the hyperparameter tuning was performed using BO in a visual field defect classification problem.

Inspired by previous works [12,13,14], this study aims to expand their investigation by incorporating the metaheuristics methods on EI function BO for multi-class classification of VF defects. The metaheuristics can be broadly classified into four categories of swarm-based algorithms [5,15], evolutionary algorithms [16,17], physics-based algorithms [16,18], and human-based algorithms [16]. These algorithms are based on the behavior of a population of social organisms. These algorithms simulate the behavior of a swarm of agents collaborating to find the best solution to an optimization problem. Previously, a metaheuristics method, the PSO method, was employed by Li et al. [5] to optimize the hyperparameter of BO in the Random Forest (RF) classifier, Adaptive Boosting (AdaBoost), and Extreme Gradient Boosting (XGBoost). A few works in medical imaging examine the classification of a specific eye disease. For instance, Omer et al. [19] combined DL and metaheuristic methods to diagnose diabetic retinopathy. In another work, Nagaraja et al. [20] proposed a metaheuristic method to optimize the Principal Component Analysis (PCA) and HHO to optimize the CNN method for detecting diabetic retinopathy. Most previous research has focused on optimizing the binary classification problem using fundus images.

Therefore, this work aims to enhance the efficiency and accuracy of multi-class image classification by integrating the BO acquisition function with the metaheuristic approach. This work incorporates the Swarm-based algorithms [5], namely Particle Swarm Optimization (PSO) [5,21], Artificial Bee Colony (ABC) Optimization [13,22], Harris Hawks Optimization (HHO) [23], and SFO [24], with the acquisition function EI in BO. The metaheuristic functions are expected to enhance the acquisition function by optimizing the Exploration (mean) and Exploitation (variance) of the posterior distribution of the objective function. These four methods were chosen because swarm-based metaheuristic methods have produced remarkable results in solving complex optimization problems [25]. Furthermore, it is due to their capability of decentralized control of search agents to explore the search environment more effectively [25]. The novelty of this work can be summarized as follows:

1. To optimize the exploration and exploitation process by incorporating with the swarm-based metaheuristic methods (PSO, ABC, HHO, and SFO) with BO default acquisition function, EI.
2. To conduct a comprehensive investigation and analysis of the performance of the acquisition function in BO when incorporated with four different metaheuristic methods against the VGGNet pre-trained models.
3. To evaluate the performance of VGGNet pre-trained models after the BO enhancement in a multi-class image classification problem for VF defects images.

This paper is organized as follows: Section 1 presents the introduction of this work and some review of previous works. Section 2 explains the collection of datasets used in this work and the process of BO enhancement using the metaheuristic method. Section 3 discusses the experimental framework for conducting the effectiveness of the metaheuristic method in BO in classifying the visual field defect pattern using transfer learning. Lastly, Section 4 presents the conclusion and potential for future research.

## 2. Methodology

### 2.1. Data Collection

The work obtained 1200 visual field (VF) images from a public dataset. The first dataset is the Humphrey 10-2 Swedish Interactive Threshold Algorithm (SITA) [26,27]. The second dataset is Humphrey 24-2 from Rotterdam Eye Hospital [28,29] and the RT_dataset from Kucur et al. [30,31]. In addition, it included 68 VF images from the Department of Ophthalmology at Universiti Sains Malaysia (USM). All datasets will go through pre-processing to improve the quality and standardization of images, which can result in more accurate and efficient analysis. The dataset will then be divided 80:10:10 between the training, validation, and testing datasets. Table 1 presents the distribution of VF Defects from collected datasets.

### 2.2. Multi-Class Classification of VF Defect Using Transfer Learning

**Figure 1:**
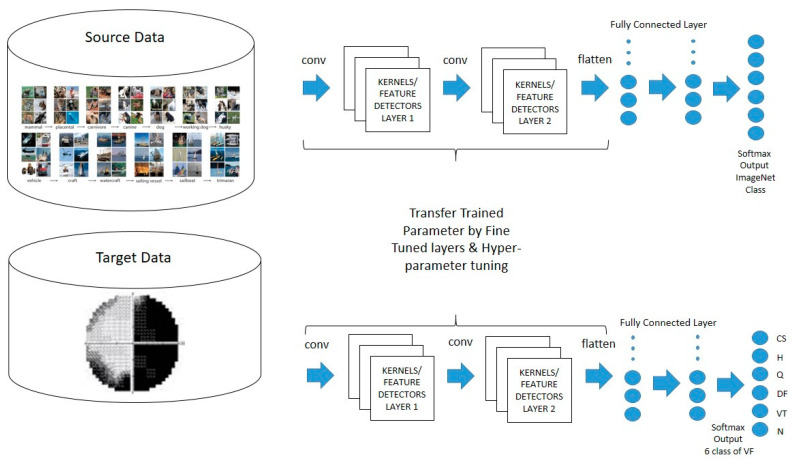
Transfer learning process on visual field defect.

In this work, the multi-class classification task of VF defect is performed by using transfer learning (TL) techniques involving four pre-trained models: VGGNet [32], ResNet [33], MobileNet [34,35], and DenseNet [36]. The ImageNet database is the source dataset utilized in these pre-trained models, which includes various eye datasets in the ImageNet collection, including black and white and face images. The knowledge acquired by the pre-trained models was then transferred to the VF classification task, enabling the model to learn more quickly and perform better with less data. Only the VGGNet model is used because of its simple architecture. The framework of transfer learning in this study is shown in Figure 1.

### 2.3. Combination of Bayesian Optimization with Metaheuristics Methods

After the transfer learning model has been built, the layers and hyperparameters of the pre-trained model will be optimized using the BO technique to identify the optimal hyperparameter for the VF defect classification task. This study investigates and analyses the effect of a different set of hyperparameters and fine-tuned layers with two different pre-trained models using a total of 11 hyperparameters to be optimized, including fine-tuning layers. Fine-tuning will be performed by training the final few layers on the VF dataset while freezing or fixing the remaining layers. The goal of fine-tuning is to leverage the learned features from the pre-trained models to improve the model’s performance on the VF classification task.

In BO, the utility functions are referred to as acquisition functions. The acquisition function helps achieve the optimum underlying function by exploring and exploiting regions where the uncertainty of the function is significant. The acquisition function is optimized to retrieve the next point for assessment [7,37,38]. Several acquisition functions have been developed to improve parameters in Bayesian to achieve the best estimation. The following are detailed descriptions of the acquisition functions used in this study:

- Expected Improvement (EI).

The EI [7,39] acquisition function is widely employed in BO because EI encourages the exploration of the search space by providing high values to points with high uncertainty. It also exploits promising regions by focusing on issues that have the potential to improve the current best solution. In addition, EI can capture the objective function’s local structure, which can be important when the function is non-convex or has multiple local optima. An example of a non-convex hyperparameter is the number of hidden layers in a neural network. Equation (1) [7] represents the EI process:

(1) $$EI\left(x\right)\equiv E\left[f\left(x\right)-f(x_{t}^{+})\right]$$

where

$x_{t}^{+}$ = the best point to observe before the next point.

*x* = default point to observe.

- Probability Improvement (PI).

The Probability Improvement (PI) acquisition function can identify the minimum value of the objective function [7]. The point where the objective function is most likely to outperform the default value is where it will be evaluated. It is equivalent to the utility function below that is connected to evaluating the objective function at a specific point with the default hyperparameters set as shown in Equation (2) [7]:

(2) $$PI\left(x\right)=P\left(f\left(x\right)\geq f\left(x+\right)\right)=\emptyset (\mu \left(x\right)-f\left(x+\right)/\sigma (x))$$

where

$f\left(x^{+}\right)$ = the max value already found.

$\mu \left(x\right)$ = the mean value of accuracy.

σ(x) = the standard deviation of accuracy.

∅() = the accumulative density function of normal distribution.

- Upper Confidence Bound (UCB).

UCB [7] is an acquisition function that maximizes or minimizes the trade-off parameter and the marginal standard deviation of the objective function rather than the objective function itself [7]. Therefore, Equation (3) [7] is employed to express the UCB process:

(3) UCBx;β=μ−+x−βσ(x)

where

β = β > 0 is a trade-off parameter.

μ(*x*) = mean of the model.

$\sigma \left(x\right)$ = standard deviation of the model.

- Lower Confidence Bound (LCB).

LCB [40,41] aims to address the random bandit dilemma by balancing exploitation and exploration. It controls the trade-off between exploitation and exploration in a manner similar to the UCB process. The LCB process is described in Equation (4) [40,41]:

(4) $$LCB\left(x\right)=-\{f\left(x\right)-\beta S(x)\}$$

where

β = the parameter managing the trade-off between exploitation and exploration.

f(*x*) = objective function.

$s\left(x\right)$ = covariance of the objective function.

The enhancement of the acquisition function will only be tested using BO’s default acquisition function, EI. The remaining acquisition functions will be used as a comparative assessment. In order to maximize the objective function, EI will be combined with four metaheuristic methods in a separate experiment setting, and the performance will be measured by the mean accuracy of BO iterations. The details of the metaheuristic method are explained below:

- Particle Swarm Optimization (PSO) [5,21] is an algorithm inspired by the behavior of a flock of birds or a school of fish. It is developed in the form of population-based stochastic optimization. In PSO, the optimal solution is obtained if the algorithm has reached convergence. If the algorithm has reached convergence, it is influenced by the particle’s position and velocity.
- Artificial Bee Colony (ABC) Optimization [13,22] contains four phases: initialization phase, employed bee phase, onlooker bee phase, and scout bee phase. Different kinds of bees can change their roles iteratively until the termination condition is met. Note that there is an associated counter for each food source. If one food source is not improved, the increment of its corresponding counter is 1; otherwise, the counter resets to 0. If the quality of a solution has not been enhanced more than the limit (present parameter), the employed bee would be transformed into a scout bee.
- Harris Hawks Optimization (HHO) [23] is the cooperative behavior in which the chasing style of Harris’ hawks in nature is called surprise pounce. HHO can reveal various chasing patterns based on the dynamic nature of scenarios and escaping patterns of the prey. The effectiveness of the HHO optimizer underwent 29 benchmark problems and several real-world engineering problems through a comparison with other nature-inspired techniques to check the optimizer’s performance.
- Sailfish Optimization (SFO) [24] is inspired by a group of hunting sailfish. This method consists of two tips of populations, the sailfish population for intensification of the search around the best so far and the sardine population for diversification of the search space. This technique indicates competitive results for improving exploration and exploitation phases, avoiding local optima, and high-speed convergence, especially on large-scale global optimization.

In Bayesian Optimization, exploration $\mu \left(x\right)$ and exploitation σ(x) are two opposing strategies used by the acquisition function to determine which set of hyperparameters to evaluate next. Exploration refers to the process of selecting hyperparameters that have a high potential for discovering new regions of the search space [42]. On the other hand, exploitation refers to the process of selecting hyperparameters that have the highest probability of improving the model’s performance based on the information gathered [42]. The main issue is that computing a natural anticipated utility function with this acquisition function is impossible. The stopping criterion is met in BO when the maximum number of evaluations or a convergence threshold is met. By incorporating a metaheuristic approach to influence the point that BO chooses, the number of points with lesser accuracy can be decreased, and the performance of BO can be increased. In addition, the number of iterations is set during BO to obtain accuracy from different sets of hyperparameters. Hence, VF defect classification will serve as the basis for measuring the BO’s performance to find the best hyperparameter.

This work observed the optimization of the EI acquisition function in BO using four swarm-based metaheuristic methods: PSO, ABC, HHO, and SFO. The metaheuristic methods optimize the mean and variance in the acquisition function to obtain many higher peak accuracies while evaluating the objective function. Here, the mean value determines the most promising point in the search space. In the meantime, the variance value is used to balance exploration and exploitation, which is extracted by the acquisition function based on the objective function, grouped as a population subject by the metaheuristic method, and used to maximize output. The mean and variance values estimated based on the observed values of the objective function at previous evaluation points are maximized using the swarm-based metaheuristic method. Finally, the mean and variance will be set as the candidate solutions that evolve to search for the optimal solution.

The pseudocode of the enhanced BO is shown in Algorithm 1. The section highlighted in bold outlines the proposed algorithm that incorporates the metaheuristics method into BO.
**Algorithm 1:** Psuedocode of the BO Enhancement: Enhance Bayesian Optimization with Metaheuristic MethodRequired: An acquisition function 1: Inputs: Bayesian Optimization process 2: While the stop criteria are not fulfilled, do the following:  3:  Select the next point to evaluate based on an acquisition function (Expected    Improvement), which balances the exploration of new points and the    the exploitation of promising areas of the search space until maximize accuracy    is obtained:
$x^{*}=\arg\underset{x\in X}{\max}f(x)$.
**4:  Calculation of EI for each set of hyperparameters and fine-tuning layers using the probabilistic model provides:**
$EI\left(x\right)=E\left[max\left(0,f\left(x\right)-f\left(x^{*}\right)\right)\right].$
**5:  Evaluate the mean *µ*(*x*) and variance**σ(x)**of the probabilistic model at x.**   **These values are estimated based on the observed values of the objective **   **function at previous evaluation points.** **6:  Initialize the population of mean *µ*(*x*) and variance**σ(x)**of the probabilistic**   **model at x.** **7:  From the set of a population of mean *µ*(*x*) and variance**σ(x),**maximize by me taheuristic method (PSO, ABC, HHO and SFO). The goal is to explore the search space and generate a diverse set of hyperparameters and fine-tuned layers solution.** **8:  Evaluate the objective function for each new set of hyperparameters and fine-tuned layers solution in the population.** **9:  Select the best hyperparameter and fine-tuned layers from the entire population** ** based on the objective function values. ** 10: End while

### 2.4. Evaluation

The performance of the four acquisition functions above was validated with VGGNet pre-trained models. Because the iteration number is set to 11, the mean accuracy (μ) value will be used to compare the 11 accuracies obtained from the experiments. The mean of the transfer learning accuracy over multiple runs of the algorithm is calculated to evaluate the accuracy of the optimization algorithm based on the following Equation (5).

(5) $$\mu =\frac{\sum_{i=1}^{N}X_{i}}{N},$$

where

N = the size of the iteration.
$x_{i}$ = each accuracy value from the BO iteration.
μ = the iteration mean.

## 3. Experimental Results and Discussion

Four different acquisition functions (EI, PI, LCB, and UCB) were initially used to optimize the pre-trained VGGNet models in BO. Then, the mean and variance of the EI acquisition function are enhanced using four metaheuristic techniques: PSO, ABC, HHO, and SFO. EI is selected for further enhancement as it is a default acquisition function for BO. The experiment was performed using KERAS and Scikit Optimize, a Python library for performing BO. The experiment was conducted on an Intel Core i7-10 processor with 8 GB of RAM and an RTX2080 graphics processing unit (GPU).

### 3.1. Validation Analyses of Pre-Trained Models Enhanced by Bayesian Optimization Based on Different Acquisition Functions

The validation accuracy of the VGG-16 and VGG-19 models using different acquisition functions in BO based on selected hyperparameters and fine-tuned layers is presented in Table 2 and Table 3. Both tables compare the validation accuracy performance of the enhanced EI acquisition function based on the metaheuristic method compared to other acquisition functions. During the experiment, 11 hyperparameters selected by BO with fine-tuned layers were considered. The initial hyperparameters and fine-tune layer were set for the first iteration. Table 2 and 3 also include the comparison results of another metaheuristic optimization algorithm, the Covariance Matrix Adaptation Evolution Strategy (CMA-ES) [12]. Loshchilov and Hutter [12] mentioned that CMA-ES has the capacity to efficiently explore a high-dimensional search space and identify an optimal objective function solution. Additionally, therefore, the validation accuracy of CMA-ES is also included as a comparison.

The majority of the hyperparameters and fine-tuned layers in Table 2 indicate a validation accuracy of 20.55%, suggesting overfitting. This issue may occur because the pre-trained model is too complex for the VF defect image as a result of their small size or lack of diversity. Another possibility is that the set of hyperparameters is too extensive and involves all network layers, resulting in a complex network architecture. In some cases, tuning all layers of a pre-trained neural network achieved high accuracy in this classification task. It was observed that BO would adjust to a slower learning rate as more layers are tuned to prevent overwriting existing knowledge in earlier layers in order to preserve features that are likely to be useful for the new task.

The proposed method, EI-SFO, from both Table 2 and Table 3 indicates the performance of EI when enhanced using SFO metaheuristic methods. SFO is used to maximize the mean and variance processes within the EI acquisition function, but its performance cannot surpass the performance of the LCB acquisition function in BO. This is because the LCB can efficiently search the hyperparameter in the search space by selecting the hyperparameter that is likely to perform well and have low uncertainty [43]. LCB selects the lowest expected value of the transfer learning and then calculates the sum of the mean and a scaled variance value. The scaling factor will control the exploration and exploration of BO, and the larger the scaling factor values will encourage more exploration. In contrast, smaller values of the scaling factor will encourage more exploitation.

On the other hand, it was observed that the SFO method maximized the mean and variance inside the EI acquisition function. The maximization of the mean and variance of EI induced the mean to select candidate solutions that are both promising and diverse. This helps the BO to explore a broader range of the search space, potentially leading to better solutions. When the mean is maximized, the surrogate model predicts that the current hyperparameter set will likely perform well. This is a sign that the optimization process exploits the most promising areas of the search space, and it can lead to faster convergence toward the optimal set of hyperparameters [43].

In contrast, when the variance is maximized, the surrogate model is uncertain about the performance of the current set of hyperparameters. This indicates that the optimization process is exploring new areas of the search space. However, it can also result in slower convergence since the optimizer may need to evaluate more hyperparameter configurations to gain enough information about the objective function. However, the maximized mean and variance can lead to exploring suboptimal regions of the search space. Consequently, the objective function must have a well-balanced mean and variance. When comparing the standard EI to EI-PSO, EI-ABC, EI-HHO, and EI-SFO on VGG-16, the incorporation of the metaheuristic method results in a significant improvement in mean accuracy evaluations.

### 3.2. Performance Analyses of Pre-Trained Models Enhanced by Bayesian Optimization Based on Different Acquisition Functions

In BO, four acquisition functions (EI, PI, UCB, and LCB) are commonly used to optimize the objective function. Therefore, these acquisition functions were tested to analyze the difference in terms of performance in the VF defect classification task. Since the VGGNet model presents the lowest performance in the default acquisition function, i.e., EI, this work focuses on optimizing this acquisition function with four metaheuristic methods (PSO, ABC, HHO, and SFO) to enhance the performance of BO.

**Figure 2:**
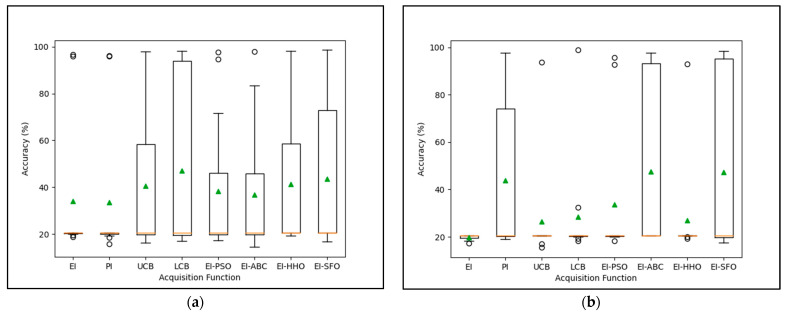
Comparison of performance accuracy distribution of VGGNet using difference acquisition function in Bayesian Optimization; (**a**) VGG-16; (**b**) VGG-19.

Figure 2a demonstrates the performance accuracy distribution across 11 iterations of the VGG-16 pre-trained model based on different acquisition functions. In this work, the number of iterations is set to 11, which is the maximum number of iterations supported by the hardware computational capability in order to achieve optimal EI performance. In the box plot green triangle represent the mean of accuracy obtain from the 11 iteration and the circle is the accuracy outlier where’s the 90% accuracy is not the majority in the 11 iteration. When optimizing hyperparameters in BO using the LCB acquisition function, LCB has shown the highest mean accuracy when compared to other acquisition functions. Without an outlier, the mean accuracy is 47.17%, with a maximum and minimum accuracy of 98.26% and 16.91%, respectively. The lower quartile value is 18.31, indicating that 25% of the obtained accuracy falls below 18.31% and 75% of the data for the upper quartile fall below 98.22. In contrast, UCB performed the worst in terms of accuracy distribution, regardless of the fact that the maximum accuracy achieved was 97.88%. The average accuracy achieved is 30.59%, with a median of 20.55% and a minimum of 16.81%.

On the other hand, Figure 2b shows the range of performance accuracy across 11 iterations of the VGG-19 pre-trained model based on different acquisition functions. Based on the box plot, the EI-SFO method outperformed other metaheuristic methods with a mean accuracy of 47.40%, a maximum accuracy of 98.34%, and a minimum accuracy of 15.50%. The lower quartile value for the EI-SFO is 19.37%. The median is 20.55%, and the upper quartile value is 95.55%. In contrast, EI has the worst performance, with a mean accuracy of 19.86%, median accuracy of 20.55%, first quartile value of 18.96%, and third quartile value of 20.55%. The maximum accuracy is 20.55%, while the minimum achievable accuracy is 17.37%.

The following Table 4 compares the performance accuracy of enhanced BO and conventional methods. For the classification of VF defects, the performance of a pre-trained model enhanced by BO using the EI-SFO method is comparable to the performance of the LCB acquisition function for VGG-16. As for VGG-19, the mean accuracy is better compared to others, while the highest accuracy is almost identical to the LCB method. The mean evaluation of VGGNet is determined by the number of iterations set during BO in order to detect VF defects with optimal accuracy. The average precision rises as the metaheuristic method optimizes the EI acquisition function. Among the enhancements to EI, the EI-SFO method achieves the highest accuracy of 98.60% for VGG-16 and 98.34% for VGG-19. The experiment also included CMA-ES, one of the acquisition functions proposed by Loshchilov and Hutter [12], that exhibits superior performance in more complex optimization problems. However, the performance of both VGGNet models for the VF classification task suggests that overfitting occurred. This could be the result of an incompatible combination of hyperparameters and fine-tuning explored by the acquisition function.

In addition to a comparative analysis of performance accuracy, a confusion matrix analysis was also conducted. The confusion matrix consists of a square matrix with dimensions equal to the number of classes in the problem. Each cell in the matrix represents the precision that belongs to a particular actual class and is predicted to belong to a particular predicted class. The diagonal elements of the matrix represent the precision of correctly classified instances for each class. The off-diagonal component represents the precision of instances that are misclassified. The darker the blue color in confusion matrix the higher the accuracy. The confusion matrix was constructed using the classification result of six types of VF defects observed in 10% of the entire dataset. The six types of VF defects include central, hemianopia, normal, quadrantanopia, superior, and tunnel defect.

**Figure 3:**
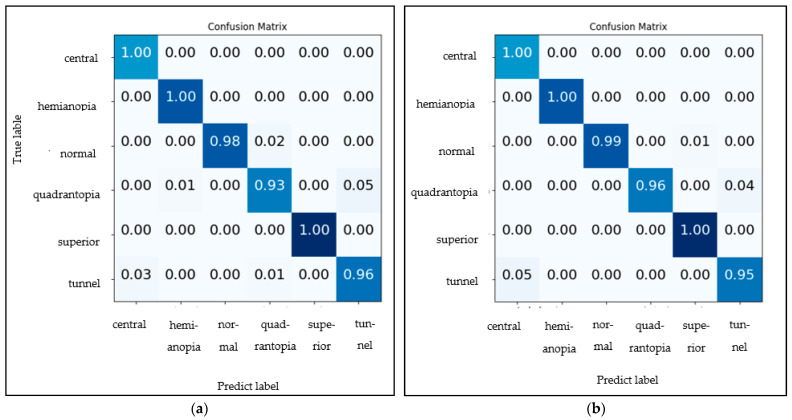
Confusion matrix for VGGNet; (**a**) VGG-16; (**b**) VGG-19.

Figure 3a,b demonstrate the performance of VGGNet after enhanced BO has been applied. Figure 3a shows that the VGG-16 model classified superior, central, and hemianopia defects with 100% accuracy. As for quadrantanopia, the obtained precision is 93%, with 5% tunnel vision and 1% hemianopia misclassification. Normal vision achieved 98% precision with 2% quadrantanopia misclassification, while tunnel vision achieved 96% precision with 3% central scotoma and 1% quadrantanopia misclassification. The overall precision obtained from six classes of visual field defects in VGG-16 is 97.83% precision. 

In Figure 3b, VGG-19 achieved a precision of 100% for central scotoma, superior, and hemianopia, 96% for quadrantanopia with 4% misclassified as tunnel vision, 99% for normal with 1% misclassified as superior, and 95% for tunnel vision with 5% misclassified as a central scotoma. The overall testing precision obtained from the six classes of visual field defects in VGG-16 is 98.33%.

## 4. Conclusions

In conclusion, the acquisition function plays a crucial role in the BO process as it determines the next set of hyperparameters to be evaluated based on the trade-off between exploration and exploitation. EI is the default acquisition function for BO, which searches for the global optimum of the objective function within the given search space. Global search algorithms can be used as an initialization strategy to sample a diverse set of points in the search space. Then, the acquisition function is used to guide the search towards more promising regions around the initial points. This helps to balance the exploration-exploitation trade-off in the search for optimal hyperparameters. In other words, the performance of the acquisition function can have a significant impact on the efficiency and effectiveness of the optimization process.

Therefore, this work proposes combining swarm-based metaheuristic methods (PSO, ABC, HHO, and SFO) with the EI acquisition function. The aim of the proposed method is to examine how the metaheuristics approach can be utilized to optimize the exploration and exploitation of EI in BO to obtain a more optimum set of hyperparameters and fine-tuned layers. The effectiveness of the proposed method was observed in a multi-class classification task which involved six different types of VF defect images. Then, a comprehensive investigation and analysis of the classification performance were conducted to determine how effectively BO optimizes the objective function in VGGNet models following the improvement of the acquisition function. These include the analyses of the validation and testing performance of the model in the VF defect classification task.

Based on the experimental result, the metaheuristic methods boost the algorithm to explore new regions of the hyperparameter and fine-tuning layers search space while searching for the optimum set of hyperparameter and fine-tuned layers to obtain a set of hyperparameter that have improved the classification accuracy. The hyperparameter tuning and fine-tuning applied shows that the feature or knowledge learned by the pre-trained model can be effectively learned by the source model by choosing the specific task features for the visual field defect pattern. The enhanced optimization processes have improved the model’s ability to identify accurate low-level features such as edges, corners and textures, as well as higher-level features such as shapes, objects, and scenes of visual field defects. 

One of the proposed methods, EI-SFO, demonstrates a promising result by obtaining high accuracy in the classification task. In comparison to other metaheuristic methods, the EI-SFO-based OB produces the highest mean accuracy for VGG-19 at 47.40%. When EI is combined with the SFO method in BO, the most significant improvement is observed when the mean accuracy for the VGG-16 model increases from 34.03% to 43.63% percent and when it increases from 19.86% to 47.40% percent for the VGG-19 model. EI-SFO-based BO is capable of producing the highest accuracy for VGG-16 and VGG-19, which is 98.60% and 98.34%, respectively. The enhanced BO of transfer learning can significantly impact medical image classification in medical diagnosis by improving model performance, enabling data-efficient model training, boosting model robustness, and saving time and resources during the hyperparameter tuning process. This can lead to more accurate and reliable classification models for medical images, which can aid in the early and accurate diagnosis of medical conditions.

Due to the constraints of our computing resources, the number of BO iterations is limited to only 11. Hence, in future, more experiments will be conducted to further analyze the performance of the acquisition function in a more significant number of BO iterations in exploring better solutions. Besides that, the combination of metaheuristics and BO to a deep learning algorithm incurs high computational complexity. Therefore, improving the algorithm’s efficiency is desirable for future investigation. In addition to the four algorithms (PSO, ABC, HHO, and SFO), more available metaheuristics algorithms such as Simulated Annealing, Ant Colony Optimization (ACO), and Flower Pollination Algorithm (FPA) can be combined with the EI acquisition function to observe their performance.

## Figures and Tables

**Table 1:** Distribution of visual field defects from collected datasets.

Type of Visual Field Defect	No. of Record
Central scotoma	204
Right/Left hemianopia	223
Right/left/upper/lower quadrantanopia	160
Tunnel vision	226
Superior/inferior defect field	181
Normal	274

**Table 2:** Validation accuracy of VGG-16 using different acquisition functions in Bayesian Optimization.

Acquisition Function	Iteration	Hyperparameter									Fine-Tuned Layer		Validation Accuracy (%)
		Feature Map	Filter Size	Activation Function	Pool Size	Optimizer	Learning Rate	Batch Size	Epoch	Dropout Rate	Upper Layer	Lower Layer	
CMA-ES [12]	1	45	1	ReLU	1	SGD	0.0001	25	49	0.3	FALSE	TRUE	20.55
	2	56	1	ReLU	2	ADAM	0.0053	20	189	0.8	FALSE	FALSE	20.55
	3	34	1	Sigmoid	1	SGD	0.0023	4	89	0.2	FALSE	TRUE	20.55
	4	53	3	Sigmoid	2	ADAM	0.0225	6	122	0.4	FALSE	FALSE	20.55
	5	51	1	ReLU	2	ADAM	0.0049	4	131	0.3	TRUE	TRUE	20.55
	6	42	3	Sigmoid	1	Adadelta	0.0711	4	127	0.3	TRUE	FALSE	20.55
	7	45	3	Sigmoid	2	Adadelta	0.0033	1	142	0.8	TRUE	TRUE	20.55
	8	61	2	ReLU	2	ADAM	0.0289	11	29	0.2	TRUE	TRUE	20.55
	9	48	2	ReLU	1	RMSprop	0.0028	16	105	0.6	FALSE	TRUE	20.55
	10	48	2	Sigmoid	2	ADAM	0.0031	17	105	0.6	FALSE	FALSE	15.67
	11	48	2	ReLU	2	RMSprop	0.0030	16	105	0.5	TRUE	TRUE	20.55
EI [39]	1	64	3	ReLU	2	ADAM	0.001	32	200	0.2	FALSE	FALSE	20.55
	2	58	2	ReLU	1	RMSprop	0.0009	24	29	0.5	TRUE	FALSE	95.97
	3	38	2	Sigmoid	1	ADAM	0.0022	10	51	0.2	TRUE	TRUE	20.55
	4	33	1	Sigmoid	1	Adadelta	0.0031	26	40	0.8	FALSE	FALSE	20.55
	5	38	2	Sigmoid	1	SGD	0.0117	13	23	0.7	TRUEs	FALSE	19.96
	6	46	2	Sigmoid	2	RMSprop	0.0019	18	34	0.6	TRUE	FALSE	20.55
	7	41	2	Sigmoid	2	ADAM	0.0003	11	76	0.9	FALSE	FALSE	20.55
	8	35	2	ReLU	1	ADAM	0.0007	29	94	0.1	FALSE	FALSE	96.74
	9	47	1	ReLU	2	RMSprop	0.0068	2	167	0.9	TRUE	TRUE	20.55
	10	56	2	ReLU	1	RMSprop	0.0264	6	97	0.4	TRUE	FALSE	18.81
	11	64	2	ReLU	2	SGD	0.0226	27	200	0.9	TRUE	TRUE	97.92
EI-PSO	1	64	3	ReLU	2	ADAM	0.001	32	200	0.2	FALSE	FALSE	20.55
	2	32	1	ReLU	2	ADAM	0.0006	9	42	0.1	FALSE	TRUE	94.75
	3	29	2	ReLU	2	Adadelta	0.0008	12	118	0.8	FALSE	FALSE	71.57
	4	17	2	Sigmoid	2	RMSprop	0.0774	10	156	0.4	TRUE	FALSE	17.16
	5	21	2	Sigmoid	1	RMSprop	0.0032	10	122	0.4	TRUE	FALSE	20.55
	6	24	2	ReLU	2	RMSprop	0.0002	7	180	0.7	FALSE	TRUE	97.80
	7	30	2	ReLU	1	RMSprop	0.0849	10	53	0.6	FALSE	FALSE	19.03
	8	32	3	Sigmoid	2	RMSprop	0.001	11	69	0.2	TRUE	FALSE	20.55
	9	25	1	Sigmoid	1	ADAM	0.0354	14	76	0.5	FALSE	TRUE	19.41
	10	18	1	Sigmoid	2	RMSprop	0.0297	9	164	0.8	TRUE	FALSE	19.96
	11	18	2	Sigmoid	2	ADAM	0.0001	14	128	0.9	FALSE	FALSE	20.55
EI-ABC	1	64	2	ReLU	2	ADAM	0.001	32	200	0.2	FALSE	FALSE	20.55
	2	19	2	ReLU	2	ADAM	0.0193	5	168	0.5	TRUE	TRUE	19.45
	3	29	1	Sigmoid	1	Adadelta	0.0231	5	141	0.8	FALSE	TRUE	20.55
	4	28	2	Sigmoid	2	ADAM	0.0213	15	30	0.6	FALSE	TRUE	15.93
	5	17	2	ReLU	2	Adadelta	0.0006	1	126	0.6	FALSE	TRUE	83.31
	6	26	2	Sigmoid	2	SGD	0.0014	6	75	0.7	FALSE	FALSE	20.00
	7	32	2	ReLU	2	SGD	0.0118	6	89	0.5	TRUE	FALSE	97.84
	8	18	1	ReLU	1	ADAM	0.0524	14	103	0.2	FALSE	TRUE	20.55
	9	26	1	ReLU	1	Adadelta	0.0009	11	184	0.5	TRUE	TRUE	71.19
	10	29	2	Sigmoid	2	RMSprop	0.0152	2	190	0.5	TRUE	TRUE	14.41
	11	22	2	ReLU	2	ADAM	0.0229	14	64	0.4	FALSE	TRUE	20.55
EI-HHO	1	64	3	ReLU	2	ADAM	0.001	32	200	0.2	FALSE	FALSE	20.55
	2	20	1	Sigmoid	1	SGD	0.0004	5	184	0.3	FALSE	FALSE	19.41
	3	30	3	Sigmoid	1	SGD	0.0005	1	193	0.4	FALSE	FALSE	20.55
	4	27	2	ReLU	2	RMSprop	0.0139	8	141	0.3	FALSE	FALSE	20.55
	5	22	2	ReLU	1	ADAM	0.0077	9	42	0.3	TRUE	FALSE	20.55
	6	23	2	ReLU	1	ADAM	0.0165	11	56	0.2	TRUE	FALSE	19.36
	7	18	2	ReLU	1	ADAM	0.0001	8	148	0.8	FALSE	TRUE	97.03
	8	26	2	Sigmoid	2	ADAM	0.0002	10	159	0.7	FALSE	FALSE	20.55
	9	18	1	Sigmoid	2	ADAM	0.0103	13	89	0.3	TRUE	TRUE	20.55
	10	24	2	ReLU	2	SGD	0.0035	2	172	0.8	FALSE	TRUE	98.26
	11	18	3	ReLU	2	RMSprop	0.0006	15	53	0.5	FALSE	TRUE	96.53
EI-SFO	1	64	3	ReLU	2	ADAM	0.001	32	200	0.2	FALSE	FALSE	20.55
	2	30	2	ReLU	1	RMSprop	0.0018	11	93	0.6	TRUE	FALSE	94.87
	3	21	2	ReLU	1	Adadelta	0.0013	8	48	0.8	FALSE	FALSE	52.92
	4	25	2	Sigmoid	1	Adadelta	0.0791	3	164	0.4	FALSE	TRUE	20.55
	5	19	1	ReLU	2	SGD	0.034	9	58	0.8	TRUE	FALSE	92.84
	6	27	3	ReLU	2	RMSprop	0.0002	15	86	0.5	TRUE	TRUE	98.60
	7	25	1	ReLU	1	ADAM	0.0466	4	150	0.5	TRUE	FALSE	19.45
	8	19	3	Sigmoid	2	ADAM	0.0055	14	169	0.1	TRUE	FALSE	16.65
	9	29	1	Sigmoid	2	RMSprop	0.0279	14	23	0.3	TRUE	FALSE	20.55
	10	18	2	Sigmoid	2	RMSprop	0.0001	3	118	0.6	TRUE	TRUE	20.55
	11	30	1	Sigmoid	1	RMSprop	0.0001	9	77	0.1	TRUE	FALSE	20.55
PI	1	64	3	ReLU	2	ADAM	0.001	32	200	0.2	FALSE	FALSE	20.55
	2	50	3	Sigmoid	1	SGD	0.0906	1	46	0.8	FALSE	FALSE	15.68
	3	63	2	ReLU	2	RMSprop	0.0158	8	127	0.8	FALSE	FALSE	20.55
	4	62	2	ReLU	1	RMSprop	0.0278	8	74	0.8	TRUE	TRUE	20.55
	5	36	3	ReLU	1	RMSprop	0.0013	14	57	0.4	TRUE	FALSE	95.85
	6	33	2	Sigmoid	1	RMSprop	0.0007	2	44	0.8	TRUE	FALSE	20.55
	7	46	2	ReLU	1	Adadelta	0.0444	19	71	0.7	FALSE	FALSE	96.23
	8	46	3	Sigmoid	2	Adadelta	0.0071	17	95	0.5	TRUE	TRUE	20.55
	9	49	2	ReLU	1	RMSprop	0.0954	3	40	0.7	TRUE	FALSE	18.52
	10	47	3	Sigmoid	1	ADAM	0.0017	19	47	0.8	FALSE	FALSE	19.36
	11	44	3	Sigmoid	1	Adadelta	0.00717	27	116	0.4	TRUE	TRUE	20.55
UCB	1	64	3	ReLU	2	ADAM	0.001	32	200	0.2	FALSE	FALSE	97.88
	2	61	2	ReLU	1	RMSprop	0.0029	22	143	0.6	FALSE	FALSE	20.55
	3	58	3	ReLU	2	SGD	0.0077	7	53	0.1	TRUE	FALSE	96.31
	4	44	3	ReLU	1	SGD	0.0075	14	162	0.2	FALSE	FALSE	96.86
	5	52	2	ReLU	1	ADAM	0.0023	1	115	0.9	FALSE	TRUE	20.55
	6	53	2	Sigmoid	2	ADAM	0.0033	5	188	0.4	TRUE	TRUE	19.07
	7	51	2	Sigmoid	2	RMSprop	0.0164	7	99	0.5	FALSE	FALSE	16.19
	8	37	1	Sigmoid	1	ADAM	0.0001	21	172	0.6	TRUE	TRUE	20.55
	9	41	3	ReLU	2	RMSprop	0.0046	8	142	0.2	TRUE	FALSE	20.55
	10	60	2	Sigmoid	2	RMSprop	0.0017	25	173	0.5	TRUE	FALSE	20.55
	11	35	3	Sigmoid	2	ADAM	0.0383	29	52	0.9	FALSE	TRUE	17.46
LCB	1	64	3	ReLU	2	ADAM	0.001	32	200	0.2	FALSE	FALSE	98.26
	2	40	3	ReLU	1	SGD	0.0313	9	14	0.3	FALSE	TRUE	94.28
	3	50	2	Sigmoid	2	ADAM	0.0498	21	167	0.1	FALSE	FALSE	18.31
	4	44	1	Sigmoid	1	ADAM	0.0010	10	34	0.9	FALSE	TRUE	20.55
	5	54	1	Sigmoid	2	RMSprop	0.0016	21	120	0.4	FALSE	TRUE	20.55
	6	52	2	Sigmoid	1	RMSprop	0.0003	17	113	0.8	TRUE	TRUE	20.55
	7	54	1	Sigmoid	2	ADAM	0.0925	5	76	0.2	TRUE	FALSE	17.33
	8	39	2	ReLU	2	ADAM	0.0002	21	100	0.9	TRUE	FALSE	98.22
	9	64	2	Sigmoid	2	RMSprop	0.0087	29	59	0.4	FALSE	FALSE	16.91
	10	59	1	Sigmoid	2	RMSprop	0.0003	26	183	0.5	FALSE	TRUE	20.55
	11	55	1	ReLU	2	Adadelta	0.0791	32	105	0.8	FALSE	TRUE	93.43

**Table 3:** Optimization of VGG-19 using different acquisition functions in Bayesian Optimization.

Acquisition Function	Iteration	Hyperparameter									Fine-Tuned		Validation Accuracy (%)
		Feature Map	Filter Size	Activation Function	Pool Size	Optimizer	Learning Rate	Batch Size	Epoch	Dropout Rate	Upper Layer	Lower Layer	
CMA-ES [12]	1	37	2	ReLU	2	Adadelta	0.0001	3	192	0.4	TRUE	FALSE	20.55
	2	48	1	ReLU	2	RMSprop	0.0528	7	92	0.3	FALSE	TRUE	17.80
	3	42	3	ReLU	2	RMSprop	0.0193	3	79	0.8	FALSE	FALSE	20.55
	4	63	3	ReLU	2	RMSprop	0.0002	2	77	0.4	FALSE	TRUE	97.03
	5	58	2	Sigmoid	2	RMSprop	0.0436	14	173	0.3	TRUE	TRUE	17.58
	6	44	3	Sigmoid	1	RMSprop	0.0026	1	25	0.3	FALSE	TRUE	17.58
	7	32	1	Sigmoid	2	Adadelta	0.0016	7	13	0.5	TRUE	TRUE	20.55
	8	63	2	ReLu	2	RMSprop	0.0006	13	37	0.4	TRUE	TRUE	93.64
	9	48	2	Sigmoid	2	RMSprop	0.0041	16	105	0.7	FALSE	TRUE	20.55
	10	48	2	ReLU	1	ADAM	0.0023	16	105	0.4	FALSE	TRUE	20.55
	11	48	2	ReLU	1	Adadelta	0.0037	16	105	0.6	TRUE	FALSE	92.16
EI [39]	1	64	3	ReLU	2	ADAM	0.001	32	200	0.2	FALSE	FALSE	20.55
	2	39	2	Sigmoid	1	SGD	0.0041	32	60	0.4	TRUE	TRUE	19.11
	3	62	2	ReLU	1	ADAM	0.0019	3	110	0.8	TRUE	FALSE	20.55
	4	39	2	Sigmoid	1	RMSprop	0.0015	31	99	0.6	TRUE	TRUE	20.55
	5	44	3	ReLU	1	RMSprop	0.0724	27	90	0.3	FALSE	FALSE	19.96
	6	32	2	Sigmoid	1	ADAM	0.0003	1	154	0.4	TRUE	TRUE	20.55
	7	36	1	ReLU	2	RMSprop	0.0874	18	58	0.7	FALSE	TRUE	18.22
	8	52	3	Sigmoid	2	RMSprop	0.0846	3	97	0.8	TRUE	FALSE	17.37
	9	55	2	ReLU	1	RMSprop	0.0029	10	172	0.8	TRUE	TRUE	20.55
	10	53	2	Sigmoid	1	ADAM	0.0001	27	142	0.6	FALSE	TRUE	20.55
	11	51	1	Sigmoid	1	ADAM	0.0019	4	135	0.7	TRUE	TRUE	18.52
EI-PSO	1	64	3	ReLU	2	ADAM	0.001	32	200	0.2	FALSE	FALSE	20.55
	2	24	2	Sigmoid	2	ADAM	0.0068	4	53	0.2	FALSE	TRUE	18.26
	3	31	3	Sigmoid	2	Adadelta	0.0532	5	72	0.6	FALSE	TRUE	20.55
	4	21	1	Sigmoid	1	ADAM	0.0453	11	53	0.3	FALSE	TRUE	19.96
	5	24	1	ReLU	2	ADAM	0.0001	7	65	0.9	FALSE	FALSE	92.67
	6	26	3	ReLU	1	Adadelta	0.0019	9	192	0.2	FALSE	TRUE	95.64
	7	31	1	ReLU	2	Adadelta	0.0003	14	67	0.6	FALSE	TRUE	20.55
	8	21	1	Sigmoid	1	SGD	0.0004	4	147	0.3	TRUE	FALSE	20.55
	9	30	1	Sigmoid	1	RMSprop	0.0002	5	45	0.8	FALSE	FALSE	20.55
	10	18	3	ReLU	1	ADAM	0.0006	4	82	0.7	FALSE	FALSE	20.55
	11	28	3	ReLU	2	RMSprop	0.06234	12	174	0.2	FALSE	TRUE	19.96
EI-ABC	1	64	3	ReLU	2	ADAM	0.001	32	200	0.2	FALSE	FALSE	97.63
	2	21	2	Sigmoid	2	ADAM	0.0005	1	114	0.8	TRUE	TRUE	20.55
	3	25	2	ReLU	2	RMSprop	0.0001	7	14	0.7	TRUE	TRUE	94.19
	4	19	2	Sigmoid	2	RMSprop	0.0016	14	112	0.4	FALSE	TRUE	20.55
	5	17	3	Sigmoid	1	ADAM	0.0005	13	116	0.4	TRUE	FALSE	20.55
	6	26	1	Sigmoid	2	ADAM	0.0017	10	114	0.6	TRUE	FALSE	20.55
	7	32	2	Sigmoid	2	RMSprop	0.0003	8	144	0.9	FALSE	TRUE	20.55
	8	21	2	Sigmoid	2	Adadelta	0.0001	2	78	0.8	FALSE	FALSE	20.55
	9	23	1	ReLU	2	ADAM	0.0015	15	131	0.8	TRUE	FALSE	94.53
	10	16	3	Sigmoid	2	RMSprop	0.0018	14	169	0.2	FALSE	TRUE	20.55
	11	17	2	ReLU	1	RMSprop	0.0001	11	16	0.9	FALSE	TRUE	92.12
EI-HHO	1	64	3	ReLU	2	ADAM	0.001	32	200	0.2	FALSE	FALSE	20.55
	2	28	1	ReLU	1	ADAM	0.0056	4	174	0.6	FALSE	TRUE	20.55
	3	25	3	ReLU	1	SGD	0.0047	2	27	0.8	FALSE	TRUE	92.88
	4	19	2	ReLU	2	RMSprop	0.0053	4	76	0.23	TRUE	TRUE	20.55
	5	18	2	ReLU	1	ADAM	0.0312	12	94	0.7	TRUE	TRUE	20.00
	6	20	1	ReLU	2	ADAM	0.0042	6	115	0.5	FALSE	FALSE	20.55
	7	23	2	Sigmoid	2	Adadelta	0.0167	8	191	0.5	FALSE	FALSE	20.55
	8	18	2	Sigmoid	2	Adadelta	0.0007	14	52	0.7	TRUE	TRUE	20.55
	9	29	3	Sigmoid	2	RMSprop	0.0015	8	132	0.1	TRUE	TRUE	20.55
	10	22	1	Sigmoid	1	ADAM	0.0361	12	186	0.4	TRUE	FALSE	19.41
	11	26	2	Sigmoid	2	Adadelta	0.0333	10	140	0.1	TRUE	TRUE	20.55
EI-SFO	1	64	3	ReLU	2	ADAM	0.001	32	200	0.2	FALSE	FALSE	20.55
	2	20	1	ReLU	1	RMSprop	0.0343	10	124	0.7	TRUE	FALSE	20.00
	3	22	1	ReLU	1	RMSprop	0.0009	16	137	0.8	FALSE	FALSE	94.87
	4	20	2	Sigmoid	2	RMSprop	0.0768	13	134	0.8	TRUE	FALSE	18.77
	5	21	2	Sigmoid	2	SGD	0.0196	14	35	0.7	FALSE	FALSE	20.55
	6	27	3	Sigmoid	2	RMSprop	0.0080	7	191	0.7	FALSE	FALSE	17.58
	7	29	1	ReLU	2	RMSprop	0.0169	11	107	0.4	FALSE	TRUE	20.00
	8	19	2	ReLU	2	RMSprop	0.0677	12	173	0.2	FALSE	FALSE	19.37
	9	31	1	ReLU	2	ADAM	0.0005	5	111	0.5	FALSE	TRUE	95.55
	10	29	1	ReLU	1	ADAM	0.0004	5	55	0.5	TRUE	TRUE	95.89
	11	20	2	ReLU	2	ADAM	0.0003	7	107	0.6	TRUE	TRUE	98.35
PI	1	64	3	ReLU	2	ADAM	0.001	32	200	0.2	FALSE	FALSE	20.55
	2	51	3	Sigmoid	1	RMSprop	0.0007	14	83	0.7	TRUE	TRUE	20.55
	3	48	3	Sigmoid	1	RMSprop	0.0678	16	108	0.3	TRUE	TRUE	18.98
	4	35	1	ReLU	2	Adadelta	0.0001	8	147	0.5	TRUE	TRUE	20.55
	5	44	2	ReLU	1	RMSprop	0.0002	21	199	0.8	FALSE	FALSE	97.67
	6	50	2	ReLU	2	SGD	0.001	13	70	0.1	TRUE	TRUE	76.99
	7	54	3	ReLU	1	ADAM	0.001	4	41	0.2	TRUE	TRUE	97.12
	8	47	1	ReLU	2	SGD	0.0013	13	65	0.7	FALSE	TRUE	71.31
	9	58	1	ReLU	2	ADAM	0.0027	5	145	0.3	FALSE	FALSE	20.55
	10	40	2	Sigmoid	1	ADAM	0.0017	20	25	0.8	TRUE	TRUE	19.96
	11	40	1	ReLU	1	RMSprop	0.0372	29	23	0.9	FALSE	TRUE	19.41
UCB	1	64	3	ReLU	2	ADAM	0.001	32	200	0.2	FALSE	FALSE	20.55
	2	46	1	Sigmoid	1	RMSprop	0.0255	14	195	0.7	TRUE	TRUE	16.95
	3	54	2	ReLU	1	ADAM	0.0028	5	112	0.5	TRUE	FALSE	20.55
	4	40	1	ReLU	2	RMSprop	0.0004	5	37	0.3	FALSE	TRUE	93.64
	5	57	1	Sigmoid	1	ADAM	0.0036	26	171	0.7	TRUE	TRUE	20.55
	6	55	1	Sigmoid	2	ADAM	0.0006	16	160	0.1	TRUE	TRUE	20.55
	7	63	3	ReLU	2	ADAM	0.0022	5	129	0.8	TRUE	TRUE	20.55
	8	55	1	Sigmoid	2	ADAM	0.0002	18	180	0.4	FALSE	TRUE	20.55
	9	42	3	Sigmoid	2	ADAM	0.0249	3	92	0.4	TRUE	FALSE	15.47
	10	50	3	ReLU	1	ADAM	0.0022	14	112	0.8	TRUE	FALSE	20.55
	11	40	1	ReLU	2	RMSprop	0.0004	5	37	0.3	FALSE	TRUE	93.60
LCB	1	64	3	ReLU	2	ADAM	0.001	32	200	0.2	FALSE	FALSE	20.55
	2	48	2	ReLU	2	SGD	0.0116	4	63	0.6	FALSE	TRUE	98.00
	3	39	2	Sigmoid	2	ADAM	0.0016	19	151	0.6	TRUE	TRUE	19.36
	4	53	2	Sigmoid	1	ADAM	0.0312	19	172	0.9	FALSE	TRUE	18.18
	5	64	2	ReLU	2	SGD	0.0002	10	16	0.2	TRUE	TRUE	20.55
	6	59	1	Sigmoid	2	RMSprop	0.0002	11	130	0.8	TRUE	TRUE	20.55
	7	34	2	Sigmoid	2	Adadelta	0.0247	13	120	0.7	TRUE	FALSE	20.55
	8	33	2	Sigmoid	2	RMSprop	0.0003	6	56	0.6	TRUE	FALSE	20.55
	9	38	2	Sigmoid	2	ADAM	0.0042	16	95	0.2	FALSE	FALSE	19.96
	10	34	3	ReLU	2	RMSprop	0.0056	16	161	0.2	TRUE	FALSE	20.55
	11	52	1	ReLU	1	SGD	0.0004	27	110	0.6	TRUE	TRUE	32.46

**Table 4:** Comparison of performance accuracy of enhanced BO and existing methods.

Acquisition Function	Transfer Learning Model	Mean Accuracy (%)	Max Accuracy (%)	Min Accuracy (%)
CMA-ES [12]	VGG-16	20.10	20.55	15.67
	VGG-19	39.87	97.03	17.58
EI	VGG-16	34.03	97.92	18.81
	VGG-19	19.86	20.55	17.37
EI-PSO	VGG-16	38.35	97.80	17.16
	VGG-19	33.62	95.64	17.54
EI-ABC	VGG-16	36.76	97.84	14.41
	VGG-19	47.48	97.63	20.55
EI-HHO	VGG-16	41.46	98.26	19.36
	VGG-19	26.97	92.88	19.41
**EI-SFO**	**VGG-16**	**43.63**	**98.60**	**16.65**
	**VGG-19**	**47.40**	**98.34**	**15.51**
PI	VGG-16	33.54	96.23	15.68
	VGG-19	43.97	97.66	18.98
UCB	VGG-16	30.59	97.88	16.18
	VGG-19	26.40	93.64	15.47
LCB	VGG-16	47.17	98.26	16.91
	VGG-19	28.37	98.81	18.18

## Data Availability

In this study, we used publicly available VF defect images, the Rotterdam ophthalmic Data Repository [http://www.rodrep.com/longitudinal-glaucomatous-vf-data---description.html (accessed on 23 September 2021)], S1-Dataset, Github dataset [https://github.com/serifeseda/early-glaucoma-identification (accessed on 29 September 2021)], and 10-2 Humphrey SITA dataset [https://datasetsearch.research.google.com/ (accessed on 23 September 2021)]. Universiti Sains Malaysia Visual Field Defect dataset. The VF defects can be made available for reasonable requests by contacting the corresponding authors.
